# Author Correction: Longitudinal single-cell profiling reveals molecular heterogeneity and tumor-immune evolution in refractory mantle cell lymphoma

**DOI:** 10.1038/s41467-024-52013-1

**Published:** 2024-10-07

**Authors:** Shaojun Zhang, Vivian Changying Jiang, Guangchun Han, Dapeng Hao, Junwei Lian, Yang Liu, Qingsong Cai, Rongjia Zhang, Joseph McIntosh, Ruiping Wang, Minghao Dang, Enyu Dai, Yuanxin Wang, David Santos, Maria Badillo, Angela Leeming, Zhihong Chen, Kimberly Hartig, John Bigcal, Jia Zhou, Rashmi Kanagal-Shamanna, Chi Young Ok, Hun Lee, Raphael E. Steiner, Jianhua Zhang, Xingzhi Song, Ranjit Nair, Sairah Ahmed, Alma Rodriquez, Selvi Thirumurthi, Preetesh Jain, Nicolaus Wagner-Bartak, Holly Hill, Krystle Nomie, Christopher Flowers, Andrew Futreal, Linghua Wang, Michael Wang

**Affiliations:** 1 Department of Genomic Medicine, The University of Texas MD Anderson Cancer Center, Houston, TX USA; 2 Department of Lymphoma and Myeloma, The University of Texas MD Anderson Cancer Center, Houston, TX USA; 3 Department of Surgical Oncology, The University of Texas MD Anderson Cancer Center, Houston, TX USA; 4 Department of Pharmacology and Toxicology, The University of Texas Medical Branch, Galveston, TX USA; 5 Department of Hematopathology, The University of Texas MD Anderson Cancer Center, Houston, TX USA; 6 Department of Gastroenterology, Hepathology & Nutrition, The University of Texas MD Anderson Cancer Center, Houston, TX USA; 7 Department of Abdominal Imaging, The University of Texas MD Anderson Cancer Center, Houston, TX USA; 8 MD Anderson Cancer Center UTHealth Graduate School of Biomedical Sciences, Houston, TX USA; 9 Department of Stem Cell Transplantation and Cellular Therapy, The University of Texas MD Anderson Cancer Center, Houston, TX USA

Correction to: *Nature Communications* 10.1038/s41467-021-22872-z, published online 17 May 2021

The original version of this Article omitted from the author list the 7th author Qingsong Cai, who is from the ‘Department of Lymphoma and Myeloma, The University of Texas MD Anderson Cancer Center, Houston, TX, USA’. Consequently, the following was added to the Author Contributions: ‘Q.C. contributed to bioinformatics data analysis of cell lines.’ This has been corrected in both the PDF and HTML versions of the Article.

