# Supplementary material for: Longitudinal single-cell profiling reveals molecular heterogeneity and tumor-immune evolution in refractory mantle cell lymphoma
Source: Nat Commun. 2021 May 17;12:2877. doi: 10.1038/s41467-021-22872-z (PMC8128874; doi:10.1038/s41467-021-22872-z)
Supplement: Supplementary file 1 — Supplementary Information [file 41467_2021_22872_MOESM1_ESM.pdf]

Supplementary Figures

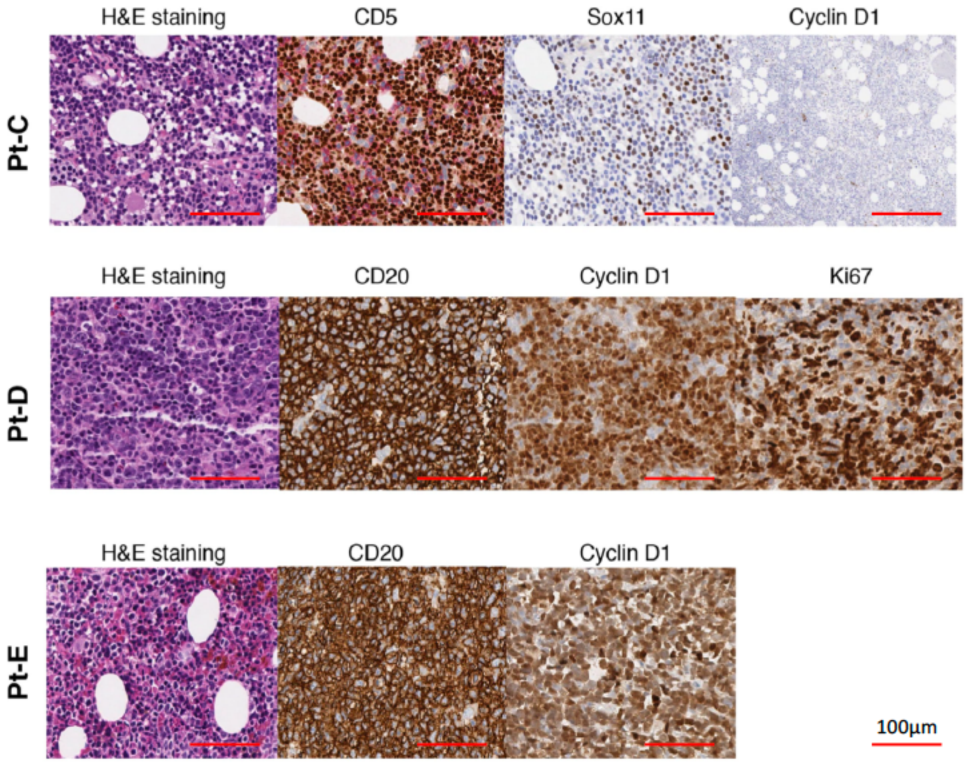

Supplementary Figure 1. H & E and immunohistochemistry staining of the bone marrow biopsies from patients B, D, E whose images were available. The images represent 5 different imaging captures (n=5).

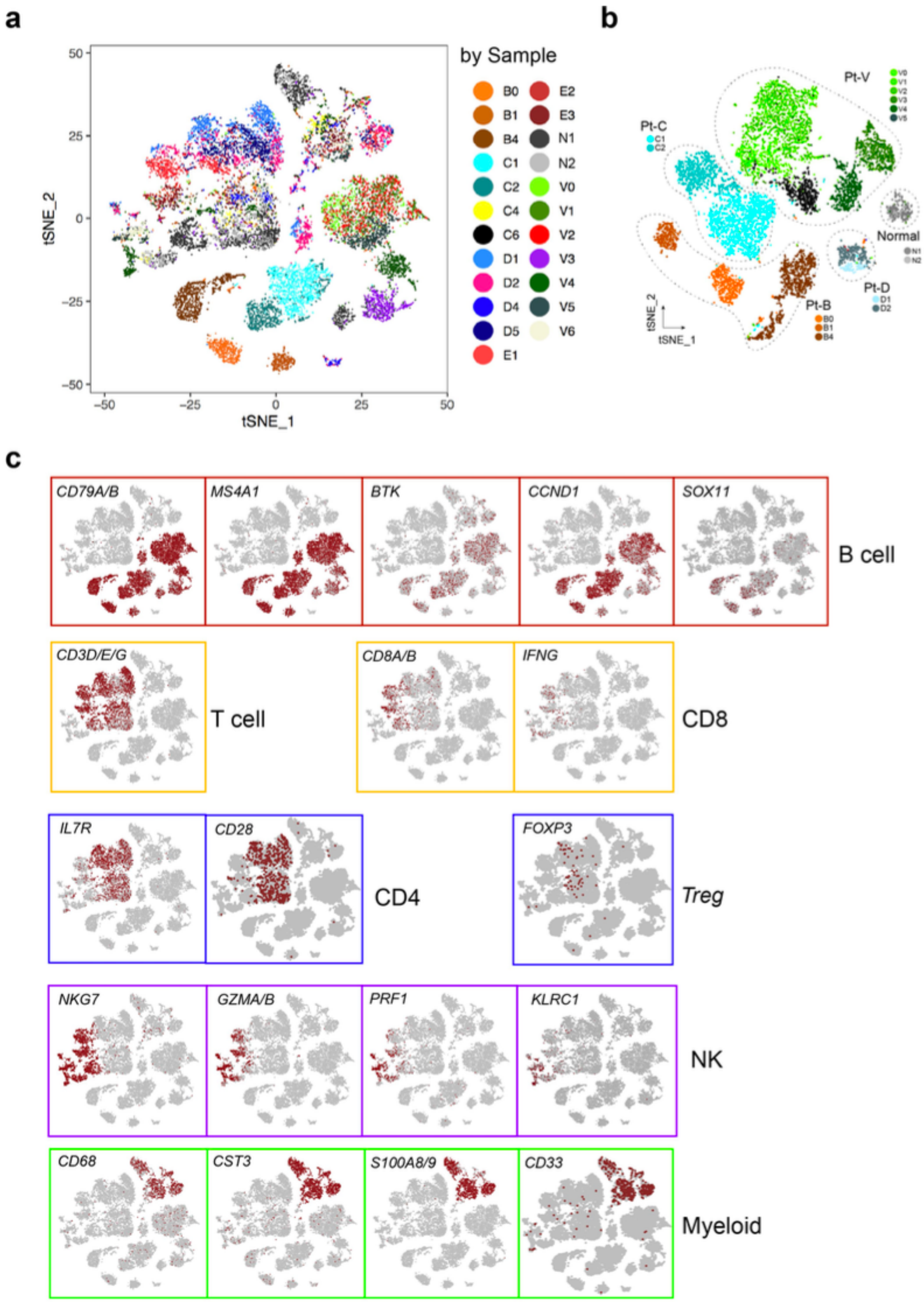

Supplementary Figure 2. Major cell clusters identified by scRNA-seq.
(**a**) An overview of the 18,794 cells that passed quality control. Each dot of the t-SNE plot represents a single cell. Cells are color-coded by their corresponding sample origin.
(**b**) An overview of the tumor cell clusters (multicolored), and normal B cells (gray). Color-coding corresponds to the sample origins.
(c) Expression of cell lineage-specific and other related markers that were used to determine major cell types.

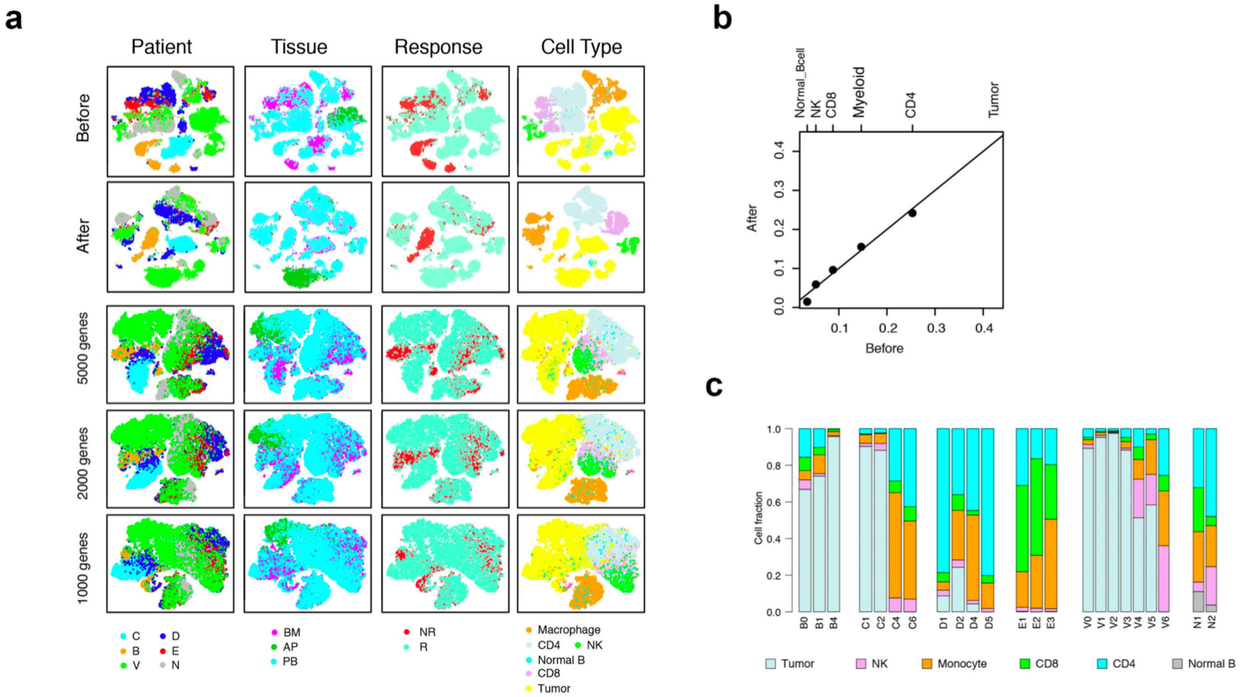

Supplementary Figure 3. Batch effects evaluation and correction.

(a) Tissue source composition by major cell types before batch effect correction.

(b) t-SNE plots before (top) and after (bottom) batch effect correction. Cells are colored (from left to right), by patient origin, tissue source, patient response status, and major cell type.

(c) The proportions of major cell types before and after batch effects correction are strongly correlated.

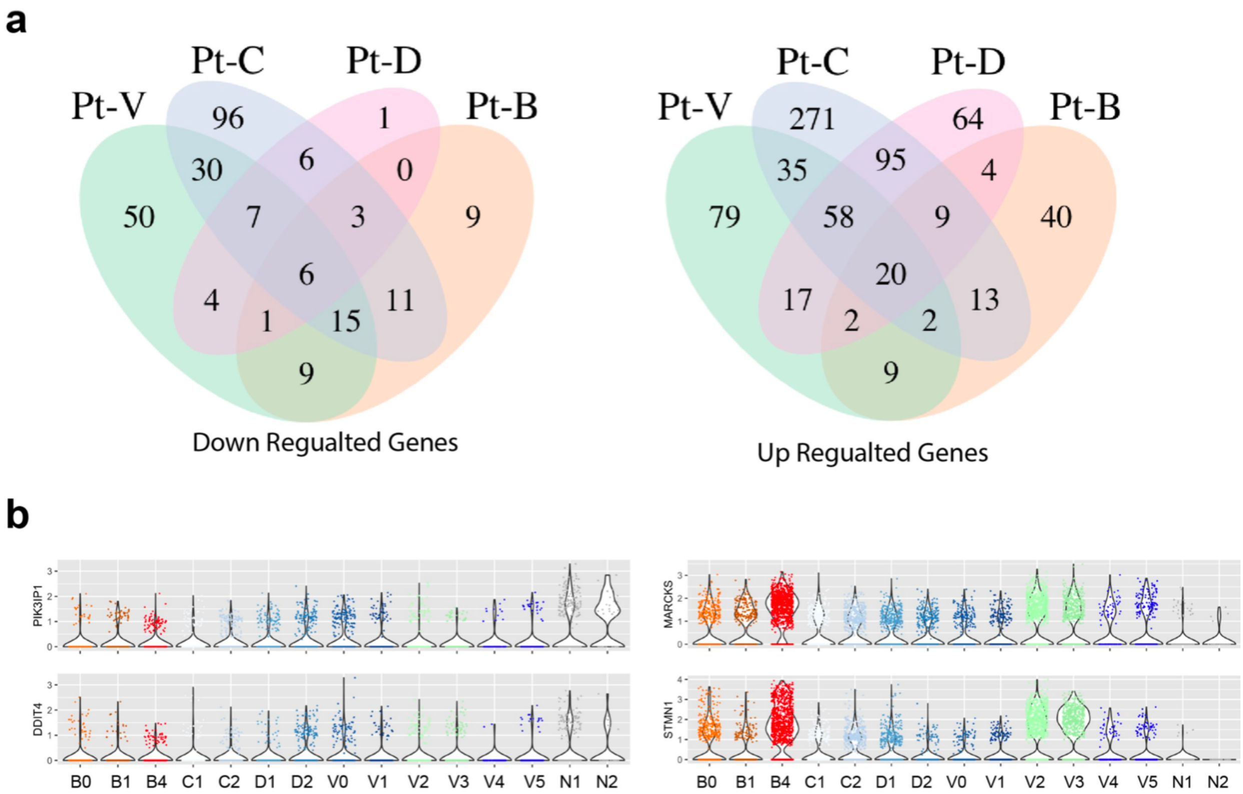

Supplementary Figure 4. Significantly differential expressed genes in MCL cells compared to normal B cells from healthy donors.

(a) Venn plot for significantly down-regulated (left) and up-regulated genes (right) across patients. For each patient, the baseline sample was included in analysis. Significant DEGs were selected by applying a cut off of log fold change > 0.5 and adjusted p value < 0.01 based on the likelihood-ratio test.

(b) Representative examples for down- and up-regulated genes identified in panel A.

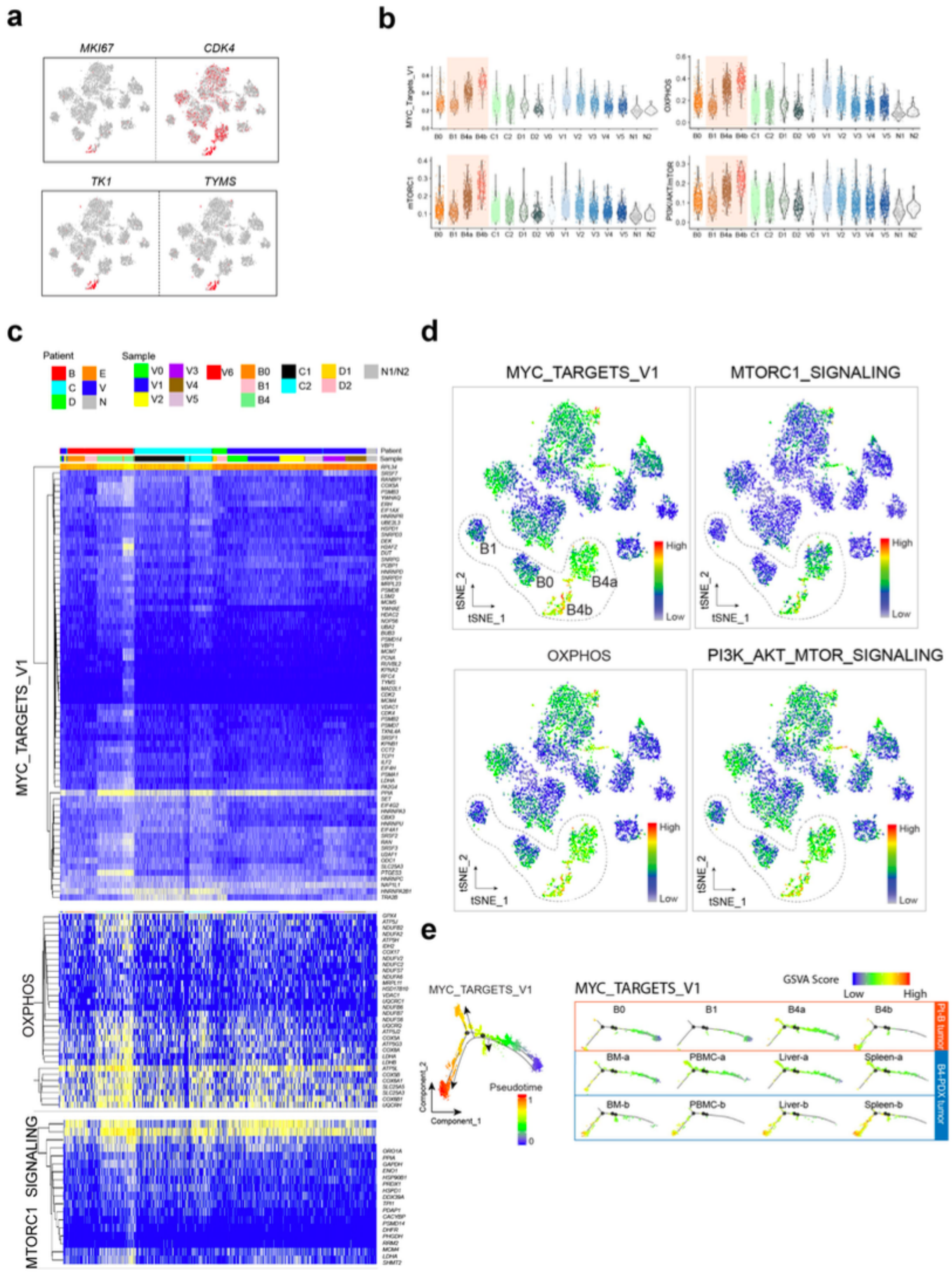

Supplementary Figure 5. The expression profiles of genes involved in key cancer hallmark pathways in MCL.

(**a**) Expression of cell cycle related genes.

(b) Representative violin plots of 4 signaling pathways selected from Fig. 2B

(**c**) Expression of key cancer hallmark pathway genes across all samples.

(**d**) Representative t-SNE of 4 signaling pathways selected from Fig. 2b.

(**e**) Developmental trajectories of tumor cell populations from patient B (B0, B1, B4a, B4b) and the tumor B4-derived PDX tumors along the pseudotime inferred by Monocle2 on the basis of MYC pathway (MYC_TARGET_V1) activity.

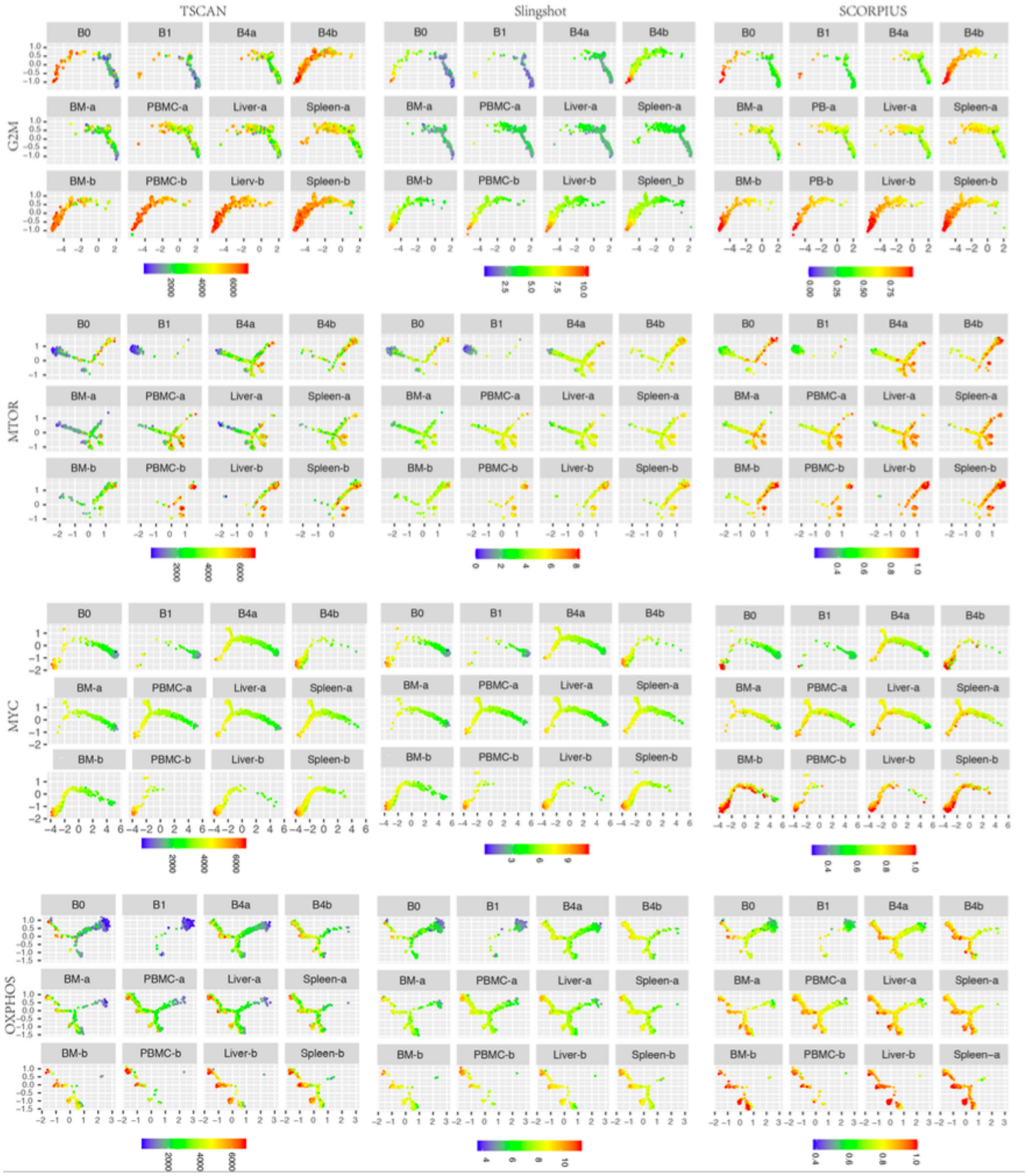

Supplemental Figure 6. Pseudotime inference using TSCAN, Slingshot and SCORPIUS on the hallmark gene sets of cell cycle, mTORC1, MYC and OXPHOS signaling, for patient B tumors and B4-derived PDX tumors. Colors are scaled by pseudotime inferred from each algorithm.

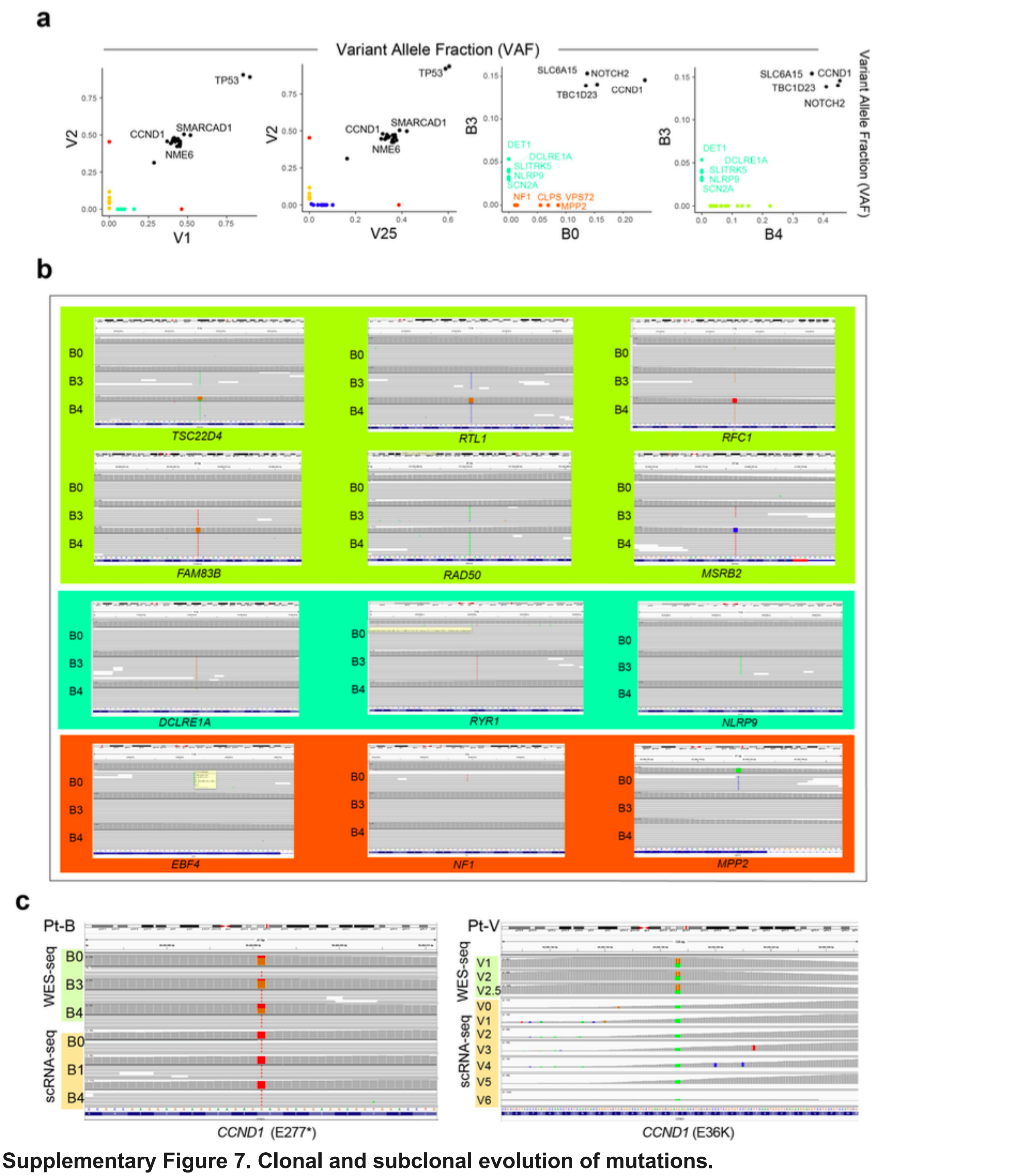

Supplementary Figure 7. Clonal and subclonal evolution of mutations.
(**a**). Changes in the variant allele fraction (VAF) of somatic mutations identified in patients V and B tumors across different sample collection time points.
(**b**) IGV view of representative somatic mutations (shown in Fig. 3e and f) identified by deep WES.
(**c**) IGV view of *CCND1* somatic mutation at DNA level by deep WES and RNA level by scRNA-seq.

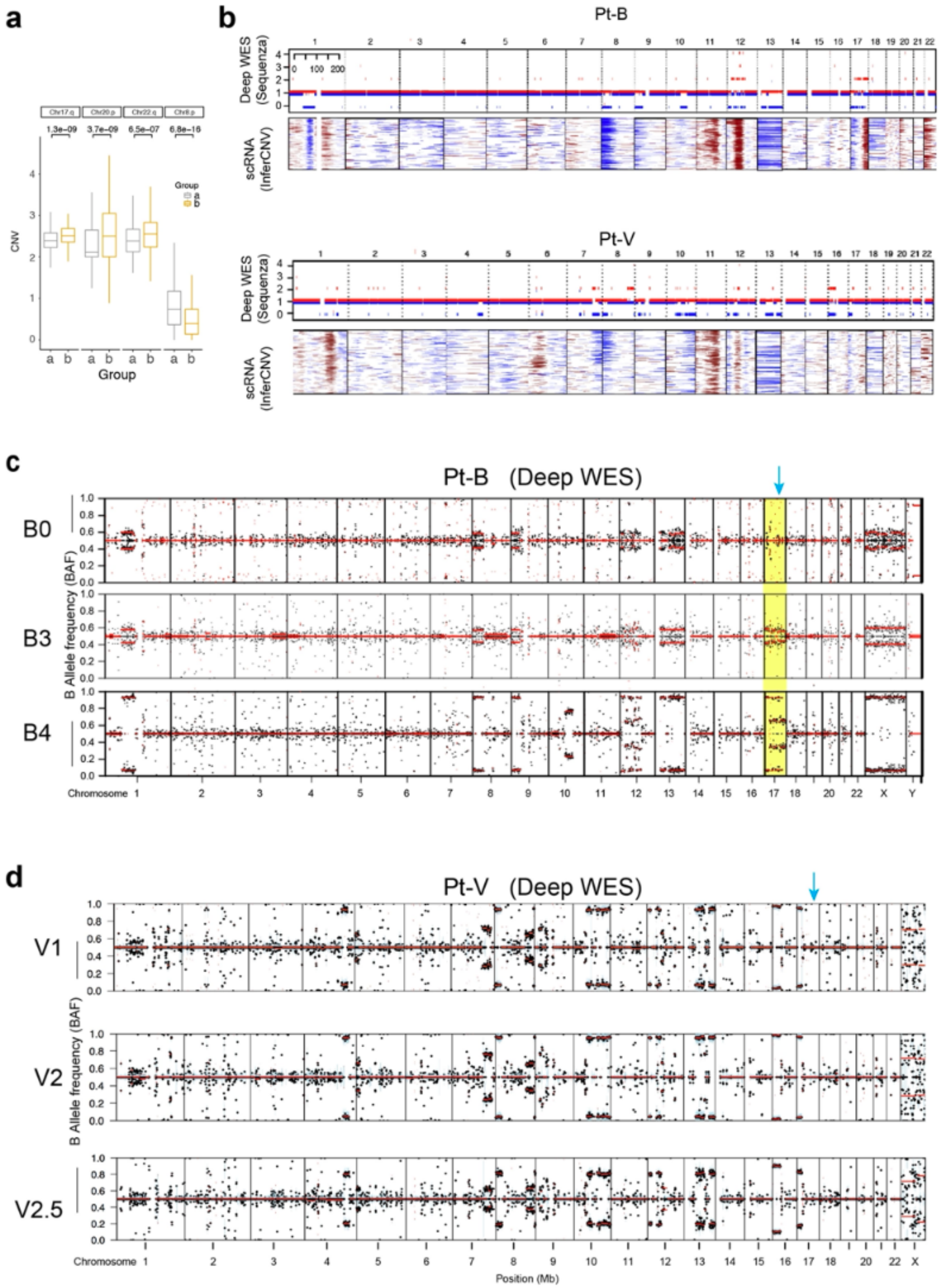

Supplementary Figure 8. Copy Number variations (CNVs) of patients B and V tumors.

(**a**) Significant chromosomal regions between B4a (n = 668 cells) and B4b (n = 248 cells). The line in the box is the median value. The bottom and top of the box are the 25th and 75th percentiles of the sample. The bottom and top of the whiskers are minimum and maximum value of sample. P value corresponds to two-side Wilcoxon Signed-rank Test.

(**b**) The allele-specific CNVs profiled by deep WES using Sequenza (top, major allele is shown in red and minor allele in blue) are concordant with calls from scRNA-seq (bottom, amplifications are shown in red and deletions are in blue).

(**c**, **d**) Copy number determined using B allele frequency (BAF) across all chromosomes with deep WES data. Each dot represents a single nucleotide polymorphism (SNP). The BAF of SNPs across patient B samples (B0, B3, B4) shows 17q gain initially at B3 stage, and was not detected in patient V samples (V1, V2, V2.5).

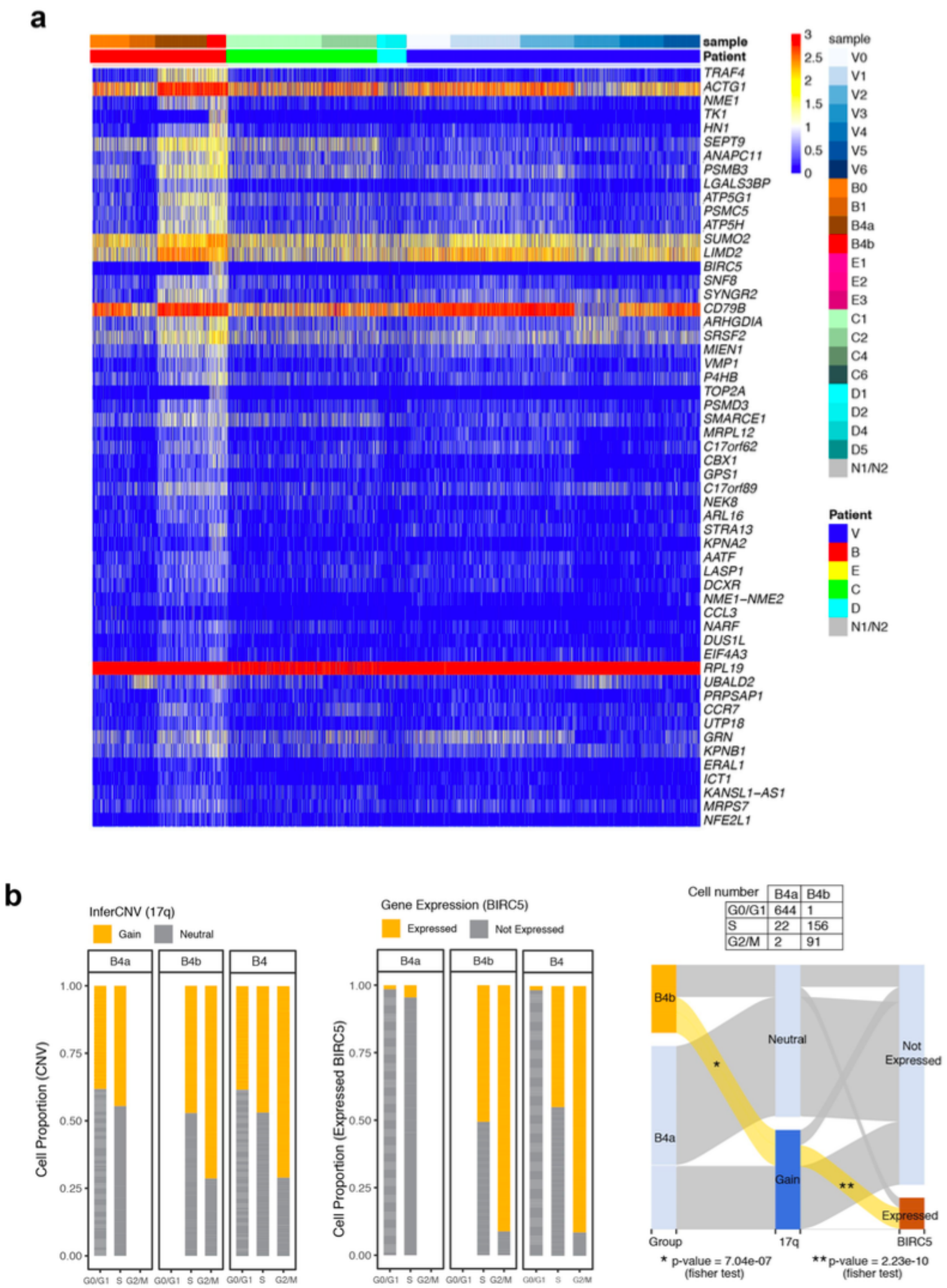

Supplementary Figure 9. 17q gain and *BIRC5* overexpression.

(**a**) Expression heatmap showing genes upregulated in the progression tumor B4 and mapped to chromosomal region 17q.

(**b**) 17q gain (left) and *BIRC5* expression (middle) in MCL cells of B4. The analysis was stratified by cell population (B4a and B4b) and cell cycle stage. The alluvial plot (right) demonstrates relationship between 17q gain and BIRC5 expression. P values are two-sided Fisher exact test.

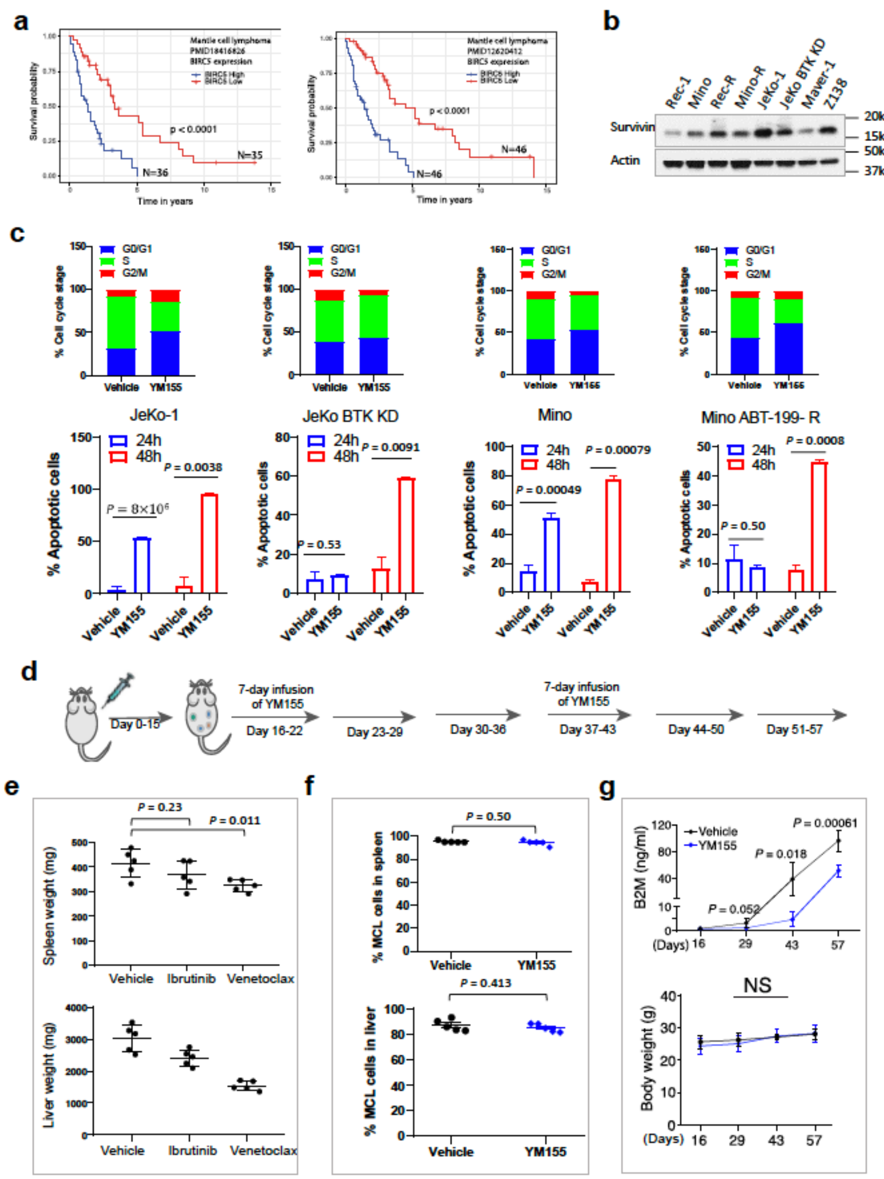

Supplementary Figure 10. Prognostic significance of survivin upregulation in MCL patient cohorts and in vitro and in vivo efficacy of the survivin inhibitor YM155 in the B4-PDX model.
(a) Prognostic significance of BIRC5 (survivin) upregulation in two MCL patient cohorts43, 44.
(b) Western blot data to detect surviving expression in 8 MCL lines. The data was reproducible by three independent experiments.
(c) YM155-induced cell cycle arrest at G0/G1 phase (top) and cell apoptosis (bottom) in MCL cell lines. The experiment was performed in triplicate (n=3). Error bars represent the standard deviation (SD). The two-sided student test was used for statistical analysis.
(d) Experiment flowchart depicting cell inoculation and drug administration steps, demonstrating in vivo efficacy of YM155 in B4-PDX model to overcome ibrutinib-venetoclax dual resistance. The mice were injected intravenously with freshly isolated B4-PDX cells from spleen of previous generation, and allowed for engraftment for 15 days. The mice carrying B4-PDX model were treated with continuous infusion of YM155 at 1.0 mg/kg on days 16-22 and days 37-43 and sacrificed on day 57 from cell inoculation.
(e) Proportion of MCL cells (hCD5+hCD20+ cells) presented in mouse spleen (top) and liver (bottom) for the dissemination cohort in YM155-treated mice in comparison to the vehicle control. The experiment was performed in 5 biological replicates (n=5). Error bars represent the standard deviation (SD). The two-sided student test was used for statistical analysis.

(f) Spleen and liver weights in response to ibrutinib and venetoclax, demonstrating that the B4 model is resistant to these agents. The experiment was performed in 5 biological replicates (n=5). Error bars represent the standard deviation (SD). The two-sided student test was used for statistical analysis.(g) B2M production in mouse serum (top) and mouse body weight (bottom) during YM155 treatment, compared to the vehicle control. The experiment was performed in 5 biological replicates (n=5). Error bars represent the standard deviation (SD). The two-sided student test was used for statistical analysis.

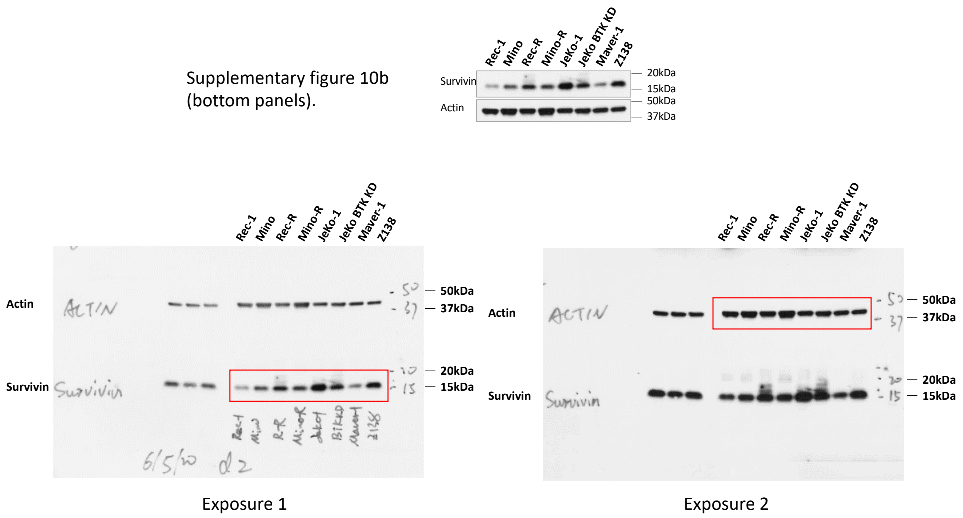

Supplementary Figure 10b. Full blot of Supplementary Figure 10b

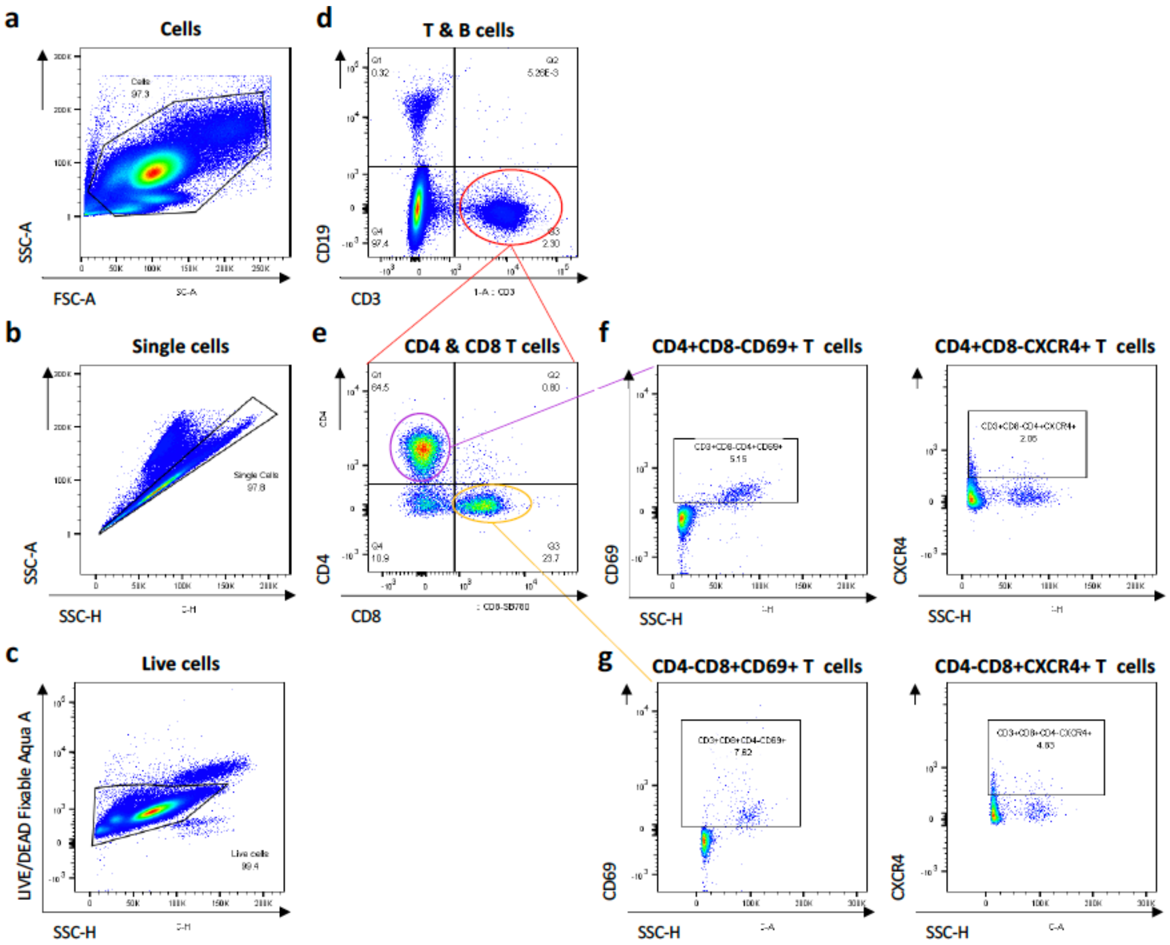

Supplementary Figure 12. FACS sequential gating/sorting strategies. (**a**) Cells were gated based on SSC-A and FSC-A. (**b**) Single cells were gated based on SSC-A and FSC-H. (**c**) Live cells were gated based on low LIVE/DEAD fixable Aqua A staining. (**d**) CD3+ T cells and CD19+ B cells were separated based on CD3 and CD19 staining. (**e**)CD4 T and CD8 T cells were separated based on CD4 and CD8 staining using the CD3+ T cell population in panel d. Data panels in **Figure 6d** were generated based on this gating. (**f-g**) CD69 (left) and CXCR4 (right) positive cells were gated based on CD69 or CDXR4 staining using the CD4 positive cells circled in purple in panel **e** (**f**) or the CD8 positive cells circled in yellow in panel **e** (**g**). Data panels in **Figure 7d** were generated based on this gating.
