# Supplementary material for: Longitudinal single-cell profiling reveals molecular heterogeneity and tumor-immune evolution in refractory mantle cell lymphoma
Source: Nat Commun. 2021 May 17;12:2877. doi: 10.1038/s41467-021-22872-z (PMC8128874; doi:10.1038/s41467-021-22872-z)
Supplement: Supplementary file 3 — Description of Additional Supplementary Files [file 41467_2021_22872_MOESM3_ESM.pdf]

Description of Additional Supplementary Files

File Name: Supplementary Data 1
Description: Clinical and histopathological characteristics of 5 MCL patients.

File Name: Supplementary Data 2
Description: Differentially expressed genes (Baseline tumor B cell vs. Health donor B cell)

File Name: Supplementary Data 3
Description: Differentially expressed genes (B4 vs. all other tumors) mapped to chromosome 17q.

File Name: Supplementary Data 4
Description: YM155 tumor volume and survival data.

File Name: Supplementary Data 5
Description: Somatic mutations identified in B & V patients by deep WES.

File Name: Supplementary Data 6
Description: SC3 cluster featured markers of patient B.

File Name: Supplementary Data 7
Description: Significant changes (NR vs. R) in ligand-receptor based cell-to-cell interactions in pre-treatment samples.

File Name: Supplementary Data 8
Description: Catalogue numbers of antibodies.
