# Supplementary material for: Longitudinal single-cell profiling reveals molecular heterogeneity and tumor-immune evolution in refractory mantle cell lymphoma
Source: Nat Commun. 2021 May 17;12:2877. doi: 10.1038/s41467-021-22872-z (PMC8128874; doi:10.1038/s41467-021-22872-z)
Supplement: Supplementary file 12 — Reporting Summary [file 41467_2021_22872_MOESM12_ESM.pdf]

nature research

Corresponding author(s): M. Wang and L. Wang

Last updated by author(s): Dec 28, 2020

Reporting Summary

Nature Research wishes to improve the reproducibility of the work that we publish. This form provides structure for consistency and transparency in reporting. For further information on Nature Research policies, see our Editorial Policies and the Editorial Policy Checklist.

Statistics

For all statistical analyses, confirm that the following items are present in the figure legend, table legend, main text, or Methods section.

n/a	Confirmed	
☐	☒	The exact sample size (n) for each experimental group/condition, given as a discrete number and unit of measurement
☐	☒	A statement on whether measurements were taken from distinct samples or whether the same sample was measured repeatedly
☐	☒	The statistical test(s) used AND whether they are one- or two-sided<br>*Only common tests should be described solely by name; describe more complex techniques in the Methods section.*
☒	☐	A description of all covariates tested
☐	☒	A description of any assumptions or corrections, such as tests of normality and adjustment for multiple comparisons
☐	☒	A full description of the statistical parameters including central tendency (e.g. means) or other basic estimates (e.g. regression coefficient) AND variation (e.g. standard deviation) or associated estimates of uncertainty (e.g. confidence intervals)
☐	☒	For null hypothesis testing, the test statistic (e.g. F, t, r) with confidence intervals, effect sizes, degrees of freedom and P value noted<br>*Give P values as exact values whenever suitable.*
☒	☐	For Bayesian analysis, information on the choice of priors and Markov chain Monte Carlo settings
☒	☐	For hierarchical and complex designs, identification of the appropriate level for tests and full reporting of outcomes
☐	☒	Estimates of effect sizes (e.g. Cohen's d, Pearson's r), indicating how they were calculated

Our web collection on statistics for biologists contains articles on many of the points above.

Software and code

Policy information about availability of computer code

Data collection	No software was used for data collection. No external data were used in this study. All single cell RNA sequencing data were generated locally in our MGL laboratory of MD Anderson Cancer Center.
Data analysis	Data analysis was performed using our standard bioinformatics pipelines, as described in the Methods. All softwares and algorithms that were applied in this study have been described previously in published literatures, which were listed in the References.<br>FlowJo, FACSDiva, CellRanger v 2.0.2; R version 3.4; Seurat v3; Monocle 2; inferCNV (v0.99); R GSVA package (version 3.9); iTALK (latest version); BWA (v0.7.5); GATK (v3.1-1); MuTect (v1.1.4); Pindel; CNTools (v1.24.0); SC3; TSCAN (v1.0), Slingshot (v0.1.3), SCORPIUS (v1.0.2), fishplot (v0.5) and sequenza (v3.0.0) package.

For manuscripts utilizing custom algorithms or software that are central to the research but not yet described in published literature, software must be made available to editors and reviewers. We strongly encourage code deposition in a community repository (e.g. GitHub). See the Nature Research guidelines for submitting code & software for further information.

Data

Policy information about availability of data

All manuscripts must include a data availability statement. This statement should provide the following information, where applicable:

- Accession codes, unique identifiers, or web links for publicly available datasets
- A list of figures that have associated raw data
- A description of any restrictions on data availability

All single cell expression data generated by this study have been deposited in the European Genome-Phenome Archive (GEA) under the accession number EGAS00001005019. We downloaded the normalized expression data of two MCL patient cohorts, in order to assess the prognostic significance of survivin upregulation in MCL. One dataset was downloaded from Rosenwald et al's website: https://llmpp.nih.gov/MCL/, and another dataset was downloaded from the

GEO database under the access code GSE10793 (https://www.ncbi.nlm.nih.gov/geo/query/acc.cgi?acc=GSE10793). 50 hallmark cancer gene sets were downloaded from MSigDB (https://www.gsea-msigdb.org/gsea/msigdb/index.jsp).

Field-specific reporting

Please select the one below that is the best fit for your research. If you are not sure, read the appropriate sections before making your selection.

☒ Life sciences ☐ Behavioural & social sciences ☐ Ecological, evolutionary & environmental sciences

For a reference copy of the document with all sections, see nature.com/documents/nr-reporting-summary-flat.pdf

Life sciences study design

All studies must disclose on these points even when the disclosure is negative.

Sample size	No sample size calculation was performed. scRNA sequencing n = 21 samples from n= 5 patients, 2 normal PBMC samples from healthy donors and 4 PDX samples. 5 mice per treatment were used for functional studies. Deep WES sequencing n = 6 samples from n=2 patient. Validation cohorts included flow cytometry 65 samples from 22 patients. In addition, we use previously published WES 14 patients and 21 patients bulk RNA-seq.
Data exclusions	No exclusion criteria was pre-established. No samples were excluded from this study. For single cell data, genes detected in <0.1% of total sequenced cells and cells where < 200 genes had nonzero counts were filtered out and not included in the analysis. Low quality cells where > 10% of the counts were derived from the mitochondrial genome were also discarded.
Replication	The experiments were performed in triplicate as indicate in Figure legends to verify the results. These data can be reproducible successfully.
Randomization	Mice were randomly assigned to different treatment groups. For the in vivo experiments, no randomization was necessary since the experiments were triplicate to verify the results.
Blinding	Investigators are blinded to specific patient identities during data analysis. The data analysis team were not involved in patient recruitment, data collection.

Reporting for specific materials, systems and methods

We require information from authors about some types of materials, experimental systems and methods used in many studies. Here, indicate whether each material, system or method listed is relevant to your study. If you are not sure if a list item applies to your research, read the appropriate section before selecting a response.

Materials & experimental systems

n/a	Involved in the study	
☐	☒	Antibodies
☐	☒	Eukaryotic cell lines
☒	☐	Palaeontology and archaeology
☐	☒	Animals and other organisms
☐	☒	Human research participants
☒	☐	Clinical data
☒	☐	Dual use research of concern

Methods

n/a	Involved in the study	
☒	☐	ChIP-seq
☐	☒	Flow cytometry
☒	☐	MRI-based neuroimaging

Antibodies

Antibodies used	Antibodies used in this work are listed in supplementary data 8.
Validation	Antibodies were validated by the manufactures and information is available on their websites. Catalogue numbers are provided in supplementary data 8.

Eukaryotic cell lines

Policy information about cell lines

Cell line source(s)	JeKo-1, JeKo BTK KD, Mino, Mino ABT-199 R, Rec-1, Rec ABT-199 R, Maver-1, and Z-138
Authentication	DNA fingerprinting
Mycoplasma contamination	Confirmed that no mycoplasma was present

Commonly misidentified lines (See ICLAC register) | No commonly misidentified cell lines was used in the study.

Animals and other organisms

Policy information about studies involving animals; ARRIVE guidelines recommended for reporting animal research

Laboratory animals	6-8 week old female NSG mice from Jackson Laboratory. Housing condition: 14/-hour light/10 hour dark cycle, ambient temperature and 40-60% humidity.
Wild animals	No wild animals were used in the study.
Field-collected samples	No field collected samples were used in the study.
Ethics oversight	MD Anderson IACUC

Note that full information on the approval of the study protocol must also be provided in the manuscript.

Human research participants

Policy information about studies involving human research participants

Population characteristics	The study population includes two females and three males aged 45 to 69. The details are listed in supplementary data 1.
Recruitment	The patients were selected for scRNA seq analysis based on patient therapeutic treatment, responses and sample availability. Due to the small cohort size, this may impact the results to be generalized. But we provided verification in larger validation cohorts.
Ethics oversight	MD Anderson Cancer center IRB

Note that full information on the approval of the study protocol must also be provided in the manuscript.

Flow Cytometry

Plots

Confirm that:

- [ ] The axis labels state the marker and fluorochrome used (e.g. CD4-FITC).
- [ ] The axis scales are clearly visible. Include numbers along axes only for bottom left plot of group (a 'group' is an analysis of identical markers).
- [ ] All plots are contour plots with outliers or pseudocolor plots.
- [x] A numerical value for number of cells or percentage (with statistics) is provided.

Methodology

Sample preparation	The patient samples were collected from peripheral blood, apheresis, or bone marrow, and subject to PBMC isolation and crypreservation. The samples were thawed and prepared in single cell suspension followed by extracelluar staining and fixation-based intracellular staining using fluorescence-conjugated antibodies.
Instrument	LSRFortessa X-20 analyzer
Software	The software used to collect the data was BD FACSDiva software and the software used to analyze the data was flowjo.
Cell population abundance	The cells was stained and analyzed without prior sorting. The cell populations was determined by cell surface markers.
Gating strategy	The cells was first gated by FSC and SSC-->single cell population-->live cell population with live/dead cell staining ->cell surface markers to determine cell populations. This is shown in supplementary figure 11.

- [x] Tick this box to confirm that a figure exemplifying the gating strategy is provided in the Supplementary Information.
